# LINC00963 affects the development of colorectal cancer via MiR-532-3p/HMGA2 axis

**DOI:** 10.1186/s12935-020-01706-w

**Published:** 2021-02-03

**Authors:** Jinjun Ye, Jidong Liu, Tao Tang, Le Xin, Xing Bao, Yukuang Yan

**Affiliations:** Department of General Surgery, Shenzhen Longgang Central Hospital, No.6082 Longgang Avenue, Longgang District, Shenzhen, 518116 Guangdong China

**Keywords:** LINC00963, Colorectal cancer, miR-532-3p, HMGA2, Biological function

## Abstract

**Background:**

LINC00963 is high-expressed in various carcinomas, but its expression and function in colorectal cancer (CRC) have not been explored. This study explored the role and mechanism of LINC00963 in CRC.

**Methods:**

The expression of LINC00963 in CRC and its relationship with prognosis were examined by starBase and survival analysis. The effects of LINC00963, miR-532-3p and HMGA2 on the biological characteristics and EMT-related genes of CRC cells were studied by RT-qPCR, CCK-8, clone formation experiments, flow cytometry, scratch test, Transwell, and Western blot. Xenograft assay and immunohistochemistry were performed to verify the effect of LINC00963 on tumor growth. The correlation among LINC00963, miR-532-3p, and HMGA2 was analyzed by bioinformatics analysis, luciferase assay, and *Pearson* test.

**Results:**

LINC00963 was high-expressed in CRC, and this was associated with poor prognosis of CRC. Silencing LINC00963 inhibited the activity, proliferation, migration, and invasion of CRC cells, MMP-3 and MMP-9 expressions, moreover, it also blocked cell cycle progression, and inhibited tumor growth and Ki67 expression. However, overexpression of LINC00963 showed the opposite effects to silencing LINC00963. LINC00963 targeted miR-532-3p to regulate HMGA2 expression. Down-regulation of miR-532-3p promoted cell proliferation, migration and invasion, and expressions of MMP-3 and MMP-9, and knockdown of HMGA2 reversed the effect of miR-532-3p inhibitor. Up-regulation of miR-532-3p inhibited the biological functions of CRC cells, and overexpression of HMGA2 reversed the miR-532-3p mimic effect.

**Conclusion:**

LINC00963 affects the development of CRC through the miR-532-3p/HMGA2 axis.

## Introduction

Colorectal cancer (CRC) is a high-risk digestive tract tumor [1]. The pathogenesis of CRC is mainly attributed to environmental and genetic factors [2]. Advances in CRC screening methods and strategies have improved the early detection of CRC [3], however, unobvious symptoms of early CRC often results in a late diagnosis and poor prognosis [4]. Therefore, understanding the pathogenesis of CRC and finding predictive biomarkers are highly necessary.

Long intergenic non-coding RNAs (lncRNAs) are defined as transcript RNAs longer than 200 nucleotides, but will not be translated into proteins. LncRNAs play important roles in several common hallmarks of cancers [5]. LncRNAs directly regulate various transcriptional, post-transcriptional and epigenetic protein-coding in the form of RNAs [6]. A growing body of research shows that lncRNAs could promote or inhibit the growth of transcriptional tumor cells [7–9]. MALAT-1, which is found to be high-expressed in CRC, stimulates high expression of SRPK 1 and then promotes the phosphorylation of SRSF1, thereby increasing the expression of AKAP-9 and ultimately promoting the proliferation and differentiation of CRC cells [10]. Ye et al. showed that high-expressed CCAT1 is closely related to the degree of invasion, tumor stage and CA199 content in CRC tumor independent of age or gender [11]. Gu et al. demonstrated that MEG3 could regulate matrix metalloproteinase-related genes [12], specifically, scientists confirmed that overexpressed MEG3 down-regulate the expressions of matrix metalloproteinases (MMP-2, MMP-9) and up-regulate the expression of its inhibitory gene TIMP-2 to inhibitthe invasion of CRC cells [12].

LINC00963, also known as MetaLnc9, has been shown to play a key role in diseases regulation. LINC00963 is involved in the progression of androgen antagonist prostate cancer to non-androgen antagonist prostate cancer through physical binding to epidermal growth factor receptor [13]. Shiao-Pieng Lee et al. [14] found that LINC00963 modulated chemosensitivity of oral cancer. Down-regulation of LINC00963 through FoxO signaling pathway inhibits renal interstitial fibrosis and oxidative stress of chronic renal failure [15]. Abnormally expressed LINC00963 has carcinogenic effects on cancers such as osteosarcoma, melanoma and prostate cancer [13, 16]. Wu et al. indicated that LINC00963 is high-expressed in hepatocellular carcinoma (HCC), and LINC00963 promotes cell proliferation and blocks the G0/G1 phase of cells via activating the PI3K/AKT signaling pathway [17]. Apart from these findings, so far, however, the expression and function of LINC00963 in CRC have not been determined. MiRNAs are important regulators of lncRNAs [18–20]. Multiple studies have shown that miRNA mutation or ectopic expression is associated with CRC [21–23]. Up-regulating miR-532-3p expression through inhibiting Wnt/β-catenin signaling pathway and enhancing chemical sensitivity has been applied in CRC molecular therapy [24]. However, whether LINC00963 functions in CRC via miR-532-3p has not been studied.

Therefore, we explored the role of LINC00963 in CRC with in vivo and in vitro experiments. In addition, the possible mechanism of LINC00963 in CRC cell regulation was examined by bioinformatics and related experiments.

## Materials and methods

### Ethics statement

Clinical experiment was approved by the ethics committee of Shenzhen Longgang Central Hospital (CW201908025). All the patients and their families signed the informed consent. The animal studies were conducted strictly following the relevant regulations of standard animal care and laboratory guidelines, and are approved by the Institutional Animal Care and Use Committee of Shenzhen Longgang Central Hospital (DJ201910003).

### Bioinformatics prediction

StarBase v2.0 (http://starbase.sysu.edu.cn/) predicted the target miRNA and showed the expression of LINC00963. The target genes of miR-532-3p were predicted by TargetScan 7.2 (http://www.targetscan.org/vert_72/), starBase v2.0, miRDB (http://mirdb.org/), miRBase (http://www.mirbase.org/). The intersection of the results generated from the above databases was visualized by Venny diagrams. Survival analysis was performed to examine the relationship between LINC00963 expression and prognosis of CRC patients.

### Tissues, cells and culture

The CRC tissues and adjacent tissues were collected from 50 CRC patients at our hospital between September 2019 and March 2020, quickly frozen and stored at -80° C after the surgical resection. The specific clinical characteristics of the 50 patients are shown in Table 1.

**Table 1:** The relationship between LINC00963 expression and clinical characteristics

Variable	n	LINC00963 expression		P value
		Increased	Preserved	
Total	50	33	17	
Sex				
Male	37	26	11	0.282
Female	13	7	6	
Age				
≤ 60	42	29	13	0.297
> 60	8	4	4	
Tumor location				
Right	17	9	8	0.068
Left	20	17	3	
Rectum	13	7	6	
Tumor size				
≤ 3 cm	18	9	9	0.073
> 3 cm	32	24	8	
Differentiation status				
Well	8	4	4	0.047
Moderate	27	16	11	
Poor	15	13	2	
Depth of invasion				
T1 + T2	15	7	8	0.059
T3 + T4	35	26	9	
Lymph node metastasis				
Absent (N0)	22	10	12	0.007
Present (N1 ~ 3)	28	23	5	
Distant metastasis				
Absent (M0)	23	12	11	0.057
Present (M1 ~ 3)	27	21	6	
TNM stage				
I	5	2	3	0.001
II	8	3	5	
III	16	9	7	
IV	21	19	2	

Normal colon cell line CCD-18Co (CRL-1459) and CRC cell lines (HCT116 (CCL-247), SW-620 (CCL-227), LOVO (CCL-229), HCT-15 (CCL-225), and SW480 (CCL-228)) were ordered from the American Type Culture Collection (ATCC, USA). All the cells were cultured in Dulbecco's Modified Eagle's Medium (DMEM) (30–2002, ATCC, USA) containing 10% Fetal Bovine Serum (30-2020) in 37 °C with 5% CO_2_.

### Cell grouping and transfection

The cell experiments consisted of three sections, and all the cells were divided into two groups according to cell line types (HCT116 cells and LOVO cells).

In the first section, the cells were grouped as follows: HCT116 cell group included Blank group (normal culture), siNC group (cells transfected with siLINC00963 negative control), and siLINC00963 (cells transfected with siLINC00963). LOVO group included Blank group (normal culture), NC group (cells transfected with LINC00963 negative control), and LINC00963 (cells transfected with LINC00963 overexpression plasmid).

In the second section, the cells were grouped as follows: HCT116 cell group, which was sub-divided into Blank group (normal culture), siNC + IC group (cells transfected with siLINC00963 negative control and miR-532-3p inhibitor control), siLINC00963 + IC group (cells transfected with siLINC00963 and miR-532- 3p inhibitor control), siLINC00963 + I group (cells transfected with siLINC00963 and miR-532-3p inhibitor), siNC + I group (cells transfected with siLINC00963 negative control and miR-532-3p inhibitor). LOVO group, which was sub-divided into Blank group (normal culture), NC + MC group (cells transfected with LINC00963 negative control and miR-532-3p mimic control), LINC00963 + MC group (cells transfected with LINC00963 overexpression plasmid and miR-532-3p mimic control), LINC00963 + M group (cells transfected with LINC00963 overexpression plasmid and miR-532-3p mimic), NC + M group (cells transfected with LINC00963 negative control and miR-532-3p mimic).

In the third section, the cells were grouped as follows: HCT116 cell group, which was sub-divided into Blank group, IC + siNC group, IC + siHMGA2 group, I + siHMGA2 group, I + siNC group. LOVO group, which was sub-divided into Blank group, MC + NC group, MC + HMGA2 group, M + HMGA2 group, M + NC group. The cells in the Blank group were normally cultured; the cells in the IC groups were transfected with miR-532-3p inhibitor control; the cells in the MC groups were transfected with miR-532-3p mimic control; the cells in the siNC and NC groups were transfected with siHMGA2 or HMGA2 negative control; the cells in the siHMGA2 group were transfected with siRNA silencing HMGA2 plasmid; the cells in the HMGA2 group were transfected with HMGA2 overexpression plasmid.

All the plasmids were commercially purchased from GenePharma Co., Ltd. (Shanghai, China). In addition, the cells were transfected strictly according to the instruction of Lipofectamine 3000 kit (L3000150, ThermoFisher, USA).

### Real-time quantitative polymerase chain reaction (RT-qPCR)

GAPDH and U6 served as controls to determine gene expressions in RT-qPCR. The expression of the target gene was calculated by 2^−ΔΔCt^ method [25]. All the primer sequences used are listed in Table 2. The extraction of total RNAs was conducted in accordance to the instructions of TRIzo Reagent (1 ml, 15596-018, Invitrogen, USA). Then, the total RNAs of each sample were quantitatively analyzed and reverse- transcribed. Reverse transcription kit (PrimeScript RT reagent Kit, RR047A, TaKaRa, Japan) was used to synthesize cDNAs. After amplified by PCR, each sample for detection was quantified using QuantStudio 3 Real-Time PCR System (ThermoFisher, USA) and a real-time quantitative PCR kit (A46113, Applied Biosystems, USA). Each sample was run in triplicate.

**Table 2:** All primer sequences in this study

ID	Forward sequence(5′-3′)	Reverse sequence(5′-3′)
LINC00963	GCCAAGGAGGGAGTTGTGGCTGC	CTGTTGCCACACCATGCACCACTCC
U6	CTCGCTTCGGCAGCACA	AACGCTTCACGAATTTGCGT
miR-532-3p	GGCTTGCAGTCGTATCCAGT	GTATCCAGTGCGTGTCGTGG
HMGA2	ACCCAGGGGAAGACCCAAA	CCTCTTGGCCGTTTTTCTCCA
GAPDH	GGAGCGAGATCCCTCCAAAAT	GGCTGTTGTCATACTTCTCATGG
DDOST	GAGACTCATTCGCTTTTCTTCCG	CTCCAAAATCTTCTACCGAAGGG
MBD1	AAGTCTTTCGCAAGTCAGGGG	TCAGCTCAACTTTGCTTCGGA
PISD	AGGACCTGCATCACTACCG	CCGATGGGCTAATCACGCTG
TARS	GGAGAAGCCGATTGGTGCT	TCAACTCAGCTCGACCTCCAT

### Cell Counting kit (CCK)-8

The pre-digested CRC cells (1 × 10^4^ cells/well) were treated with 10 μl CCK-8 reagent (C0005, TargetMol, USA) for 24 h. After the reaction, the mixed solution was read by an iMark microplate reader (BIO-RAD, USA) at 450 nm (Molecular Devices, USA) for detection. Cell viability was measured after culture at 0, 24, and 48 h.

### Cell colony formation assay

The HCT116 cells and LOVO cells were digested to prepare a cell suspension (1 × 10^3^ cells/mL). Different groups of cells in 6-well plates were incubated (37 °C, 5% CO_2_) for 2 weeks. Next, the cells were fixed with methanol (15 min), and then stained by Giemsa staining reagent (32884, Sigma, USA) for 20 min. Finally, the HCT116 cells and LOVO cells were air-dried at room temperature. The cell colon numbers were counted under a CKX53 OLYMPUS inverted biological microscope (Japan).

### Flow cytometry

The cells from each group were taken and three parallel experiments were set for each group. The cells were collected after digesting the cell suspension by trypsin and centrifuged at 4 °C for 5 min. Then the cells were resuspended in 0.5 mL PBS solution and added with 70% precooled ethanol, mixed and fixed at 4 C for 4 h. The cells were added with 500 uL PI solution (APOAF-50TST, Merck, Germany) and then operated following the instructions of the Cell Cycle. The cell cycle distribution each group were detected by CytoFLEX flow cytometer (Backman Coulter, USA). All the experiments were repeated in triplicate.

### Scratch assay

The pre-digested HCT116 cells and LOVO cells (1 × 10^5^ cells/well) were added into a 6-well plate. All the cells were incubated in a cell incubator for 24 h at 37 °C with 5% CO_2_. Next, a pipette was used to scratch a “1” on the cell plate. Serum-free medium was added and routinely incubated 48 h. The migration was detected by a microscope (CKX53, OLYMPUS, Japan) at 100 times.

### Transwell

The cells in each group were digested and counted. One day before the cell inoculation, the bottom membrane of the upper chamber (8 μm, BD Biosciences, USA) was covered by Matrix gel (354230, BD Biosciences, USA), while DMEM medium containing 10% FBS was added in the lower chamber. Cell suspension of each group was added into the upper chamber. After routine incubation for 48 h, cells remaining in the upper chamber were wiped off with a cotton swab. The invaded cells were fixed with 4% paraformaldehyde for 10 min and stained with 0.5% crystal violet (C110704-100 mg, Aladdin, China). After rinsing in tap water, the cells were observed under an inverted microscope and counted from 5 visual fields to obtain an average value.

### Western blot analysis

As the previous introduction [26], western blot was performed to detect changes in related protein expressions of HCT116 cells and LOVO cells. Specifically, total protein was extracted by Radio-Immunoprecipitation Assay (RIPA, P0013B, Beyotime, China), followed by protein concentration determination using BCA Kit (23,227, ThermoFisher, USA) and protein separation by SDS-PAGE gel. Next, the proteins were transferred to PVDF membrane transfer (IPVH00010, Millipore, USA), followed by primary antibody incubation and secondary antibody incubation. Chemiluminescence experiment suing ECL kit (SL1350-100 ml, Coolaber, China) was applied to develop the protein signal, which was photographed and then analyzed by ImageJ (version 5.0, Bio-Rad, USA). GAPDH was used as a control protein. The primary antibodies were MMP-3 (54 kDa, ab53015, 1/1000, Abcam, UK), MMP-9 (95 kDa, ab73734, 1 µg/ml), GAPDH (36 kDa, ab8245, 1/5000). Secondary antibodies were Anti-Mouse IgG (1:5000, ab205719) and Anti-Rabbit IgG (1:5000, ab205718). Original source data about western blot has been shown in Additional files 1, 2, 3 and 4.

### Dual-luciferase activity assay

Wild-type or mutant LINC00963 or HMGA2 were cloned into pmirGLO vector (E1330, Promega, USA), and the corresponding HCT116 or LOVO cells were then seeded into 24-well plates. The luciferase activity was measured by a dual-luciferase system (FR201-01, TransGen Biotech, CA) after transfection for 48 h. The cells were co-transfected with wild-type or mutant reporter vectors by miR-532-3p mimic or Blank, respectively. The experiment was repeated in triplicate.

### RNA Immunoprecipitation (RIP)

As the previous introduction [27], the combination of miR-532-3p and LINC00963 was detected by a Magna RIP Kit (Millipore, USA). Cells at 80–90% confluency were scraped off, then lysed the cells with complete RIP lysate, then incubated the cell extract with RIP buffer containing human anti-Ago2 antibody (Abcam), the negative control is normal mouse IgG (Abcam). LINC00963 and miR-532-3p levels were measured.

### Xenograft assay

BALB/c nude mice (20 ± 1 g, 6 weeks old) were purchased from Medical Experimental Animal Center of Guangdong Province. The nude mice were raised in SPF-grade experimental animal centers and provided with free access to food and water. Next, the mice were randomly divided into the following 4 groups (n = 9): siNC group [the right forelimb axilla of the mice was subcutaneously inoculated with HCT116 cells transfected with siLINC00963 lentivirus negative control (1 × 10^7^ cells/mouse)]; siLINC00963 group [the right forelimb axilla of the mice was subcutaneously inoculated with HCT116 cells transfected with siLINC00963 lentivirus (1 × 10^7^ cells/mouse)]; NC group (the mice were subcutaneously inoculated with LOVO cells (1 × 10^7^ cells/mouse) transfected with LINC00963 lentivirus negative control via the right forelimb axilla); LINC00963 group (the mice were subcutaneously inoculated with LOVO cells (1 × 10^7^ cells/mouse) transfected with LINC00963 lentivirus via the right forelimb axilla). 3–4 days after the inoculation, a successful establishment was defined when the tumor with a size of 4–5 mm can be touched at mouse axillary. During the experiment, we measured and recorded tumor size (mm^3^) on week 1, 2, 3, and 4. On week 4, the mice were anesthetized with a 0.5% sodium pentobarbital solution (P3761-25G, 50 mg/kg, intraperitoneal, Sigma, USA) to remove the tumor, photographed and weighted, and the tissues were collected.

### Histopathological and immunohistochemical (IHC) analysis

Just as others have described [28], tumor sections (4 μm in thickness) were prepared and subjected to hematoxylin and eosin (HE) staining and IHC. After deparaffinization, dehydration, and antigen retrieval, one section of each specimen was stained with hematoxylin and eosin (H&E). After dewaxing and antigen extraction, the sections were incubated with antibodies, developed by DAB solution, counterstained with hematoxylin, sealed and photographed. Each section was observed from 5 fields with an OLYMPUS CKX53 microscope, and the expression of Ki67 was measured by Image Pro Plus 6.0 software (Media Cybernetics, USA). The primary antibody and secondary antibody were Ki-67 (ab92742, Abcam), Anti-Rabbit IgG (ab205718).

### Statistical analysis

Statistical data was analyzed by SPSS 23.0 software, and expressed as mean ± SD. The line relationship between LINC00963, miR-532-3p and HMGA2 was analyzed by *Pearson* correlation coefficient. Comparisons among multiple groups were performed using one-way ANOVA, while comparisons between the two groups were performed using Student's two-tailed *t*-test. *P* < 0.05 was considered to be statistically significant.

## Results

### LINC00963 expression was significantly up-regulated in CRC cell lines and tissues, and was related to the poor prognosis and clinical characteristics of CRC patients

**Fig. 1:**
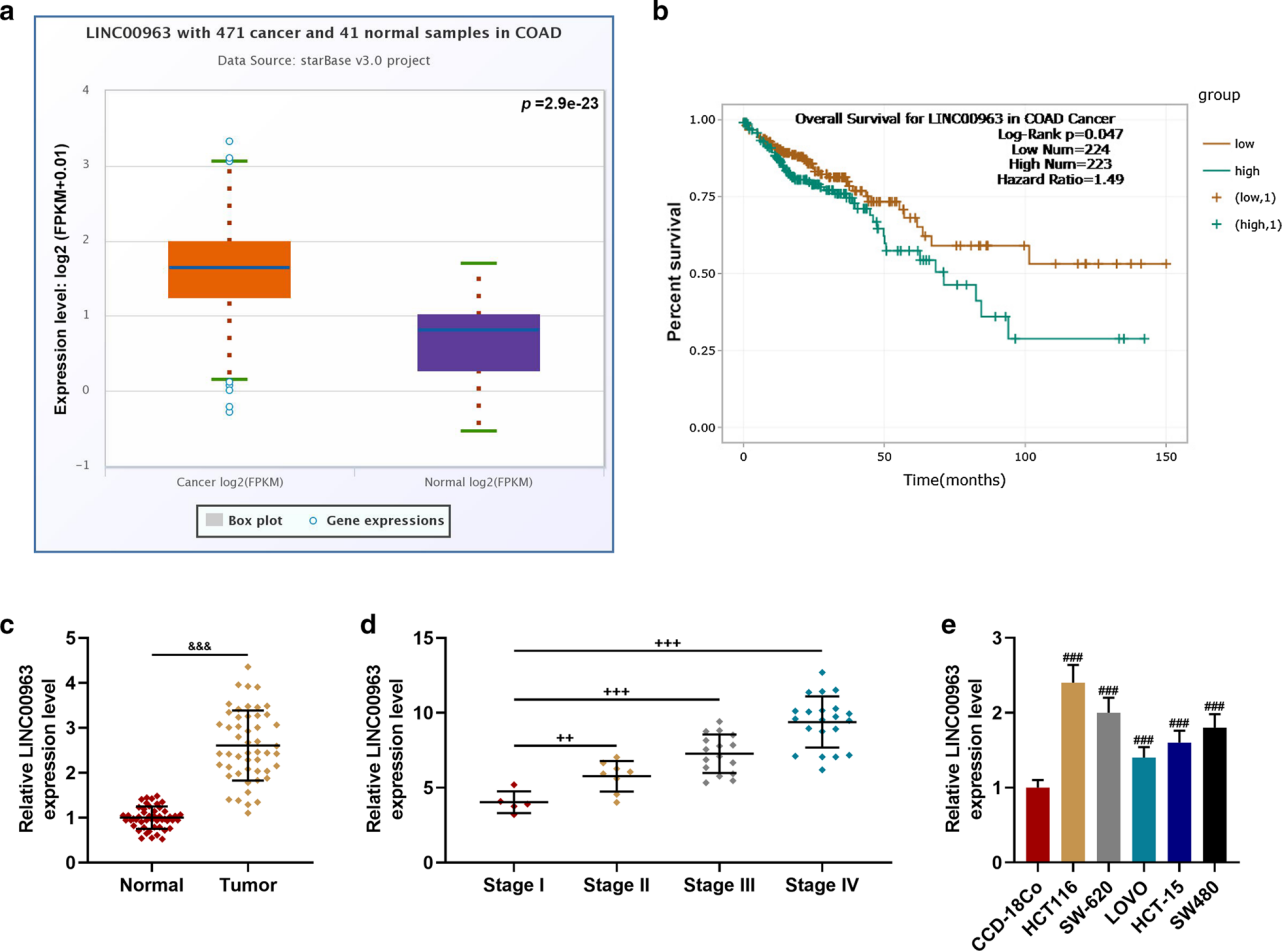
LINC00963 expression was significantly increased in colon cancer cell lines and cancer tissues, and was closely related to the poor prognosis of patients and TNM stage. **a** StarBase analysis showed that LINC00963 expression was significantly increased in colon adenocarcinoma tissues. **b** Survival analysis showed that high expression of LINC00963 was associated with poor prognosis of patients. **c** The expression of LINC00963 was up-regulated in colon cancer tissues and detected by RT-qPCR (n = 50). **d** RT-qPCR was used to detect the expression of LINC00963 in patients with colon cancer of different TNM stages. **e** The expression of LINC00963 was significantly increased in cancer cell lines, detected by RT-qPCR. GAPDH served as a control. The experiment was repeated three times independently. *RT-qPCR* real-time quantitative polymerase chain reaction. *TNM *Tumor Node Metastasis. ^&&&^*P* < 0.001 vs Normal; ^++^*P* < 0.01, ^+++^*P* < 0.001 vs Stage I; ^###^*P* < 0.001 vs CCD-18Co

StarBase analysis showed that LINC00963 expression was significantly up-regulated in colon adenocarcinoma tissues (*P* = 2.9e−23), and survival analysis indicated that high-expressed LINC00963 was associated with poor prognosis of patients (Fig. 1a, b). Thus, the expression of LINC00963 in CRC and normal tissues was further detected, and we found that LINC00963 was up-regulated in CRC tissues (*P* < 0.001, Fig. 1c). The expression of LINC00963 in CRC patients at different TNM stages was determined, and the data revealed that the expression of LINC00963 was the highest in Stage IV CRC patients (*P* < 0.01, Fig. 1d). As shown in Table 1, LINC00963 expression was related to TNM stage (*P* = 0.001), which diagnosis according to International Union Against Cancer (UICC) tumor TNM classification system [29], tumor differentiation status (*P* = 0.047), and lymph node metastasis (*P* = 0.007). The expression of LINC00963 in CRC cell lines was found to be significantly up-regulated in comparison with CCD-18Co (*P* < 0.001, Fig. 1e). Noticeably, the expression of LINC00963 was the highest in HCT116 cells and the lowest in LOVO cells.

### Effect of LINC00963 on biological characteristics of CRC cells

**Fig. 2:**
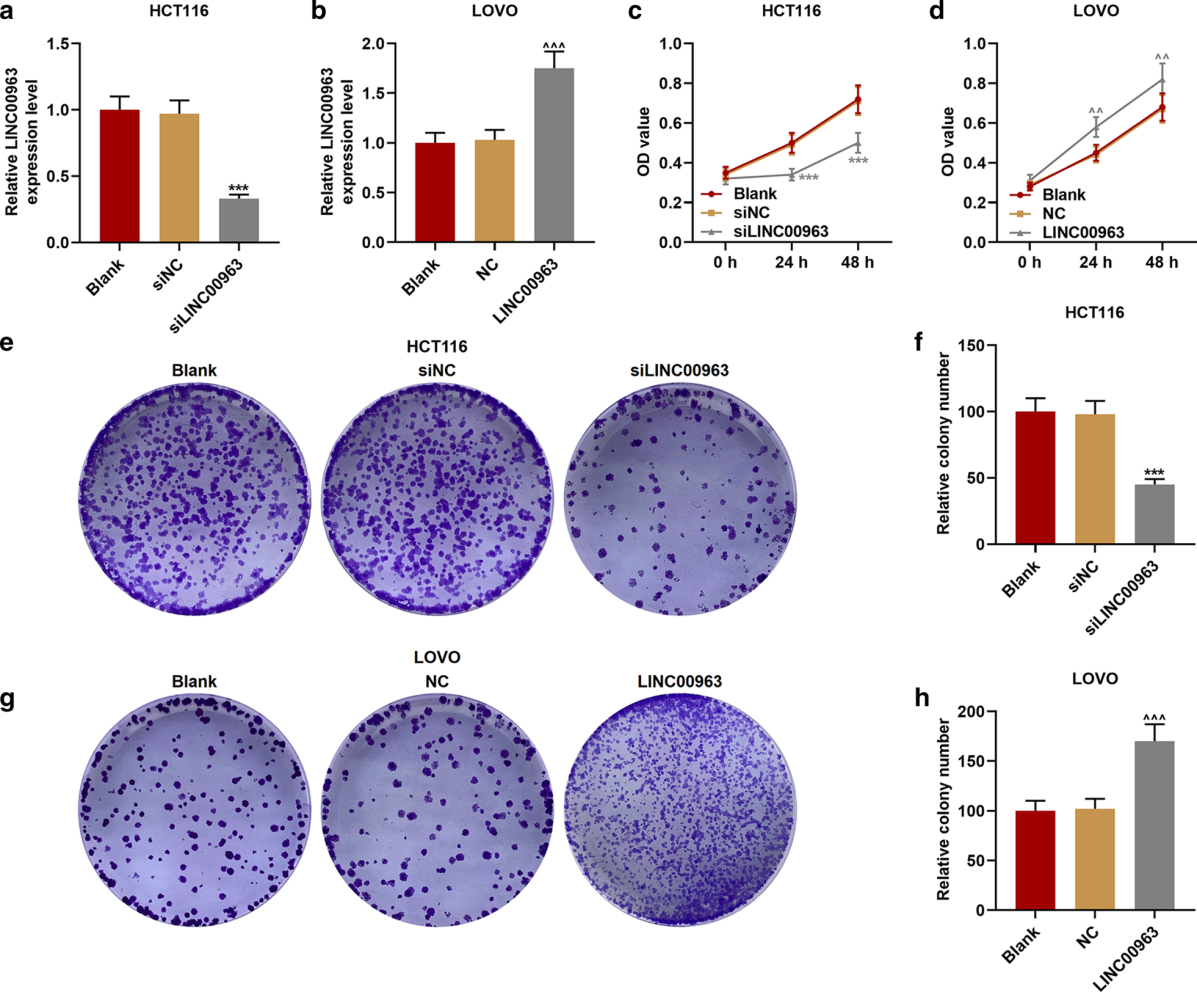
The effect of LINC00963 on the proliferation of colon cancer cells. **a** The expression of LINC00963 was decreased after transfecting siLINC00963 into HCT116 cells. **b** The expression of LINC00963 was promoted after transfection of LOVO cells with the overexpression plasmid of LINC00963. **c**, **d** CCK-8 results showed that LINC00963 overexpression increased cell activity, while silencing LINC00963 decreased cell activity. **e**–**h** Clone formation experiment was used to detect the effect of LINC00963 up-regulation or down-regulation on colon cancer cell proliferation. ^***^P < 0.001 vs siNC; ^^^^*P* < 0.01, ^^^^^*P* < 0.001vs NC

**Fig. 3:**
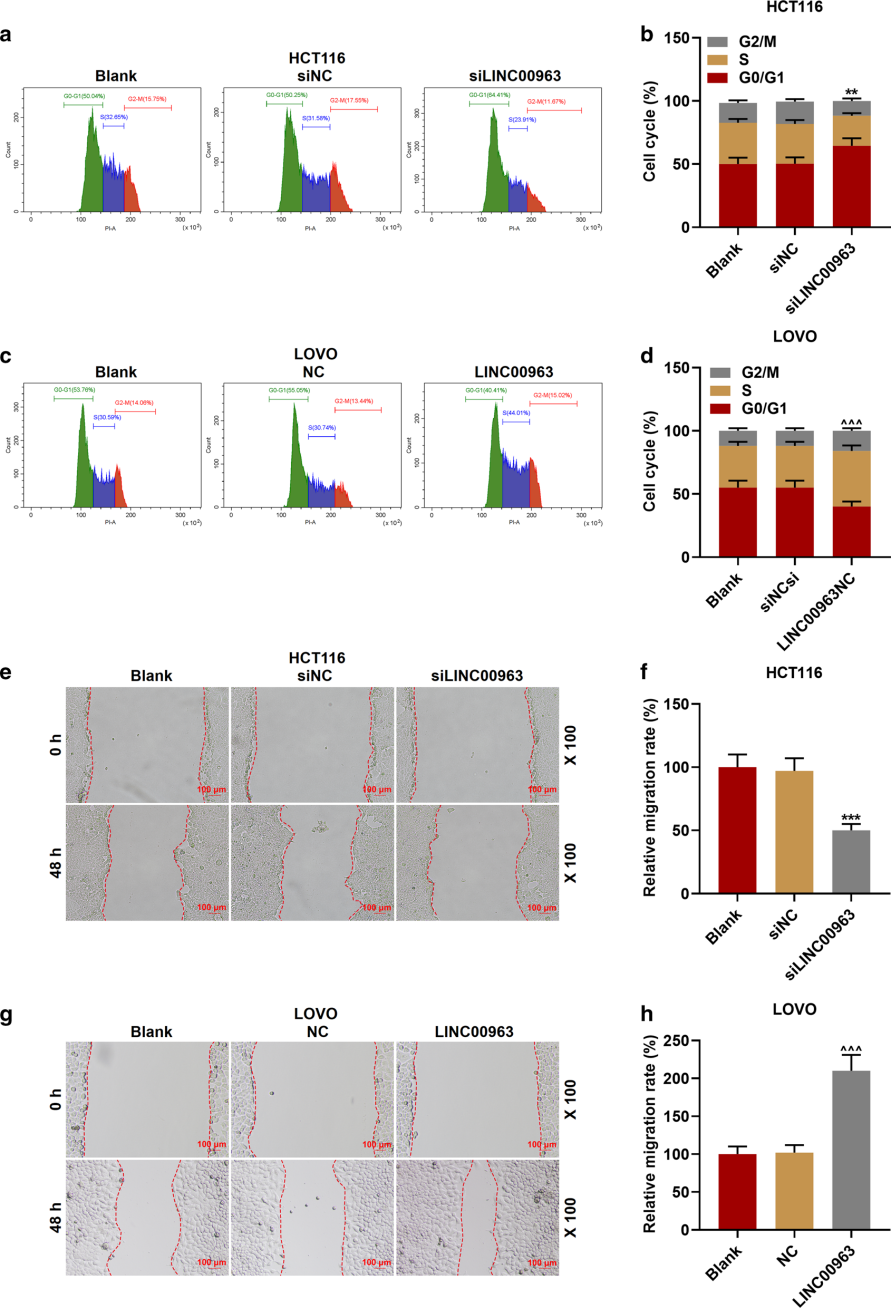
Effect of LINC00963 on colon cancer cell cycle and migration. **a**, **b** The cell cycle of the Blank, siNC, and siLINC00963 groups was measured by flow cytometry. **c**, **d** After overexpression of LINC00963, the G0/G1 phase of LOVO cells was shortened, and the S phase and G2/M phase was increased. **e**–**h** The effect of LINC00963 up-regulation or down-regulation on colon cancer cell migration was detected by scratch test. ^**^P < 0.01, ^***^P < 0.001 vs siNC; ^^^^^*P* < 0.001vs NC

**Fig. 4:**
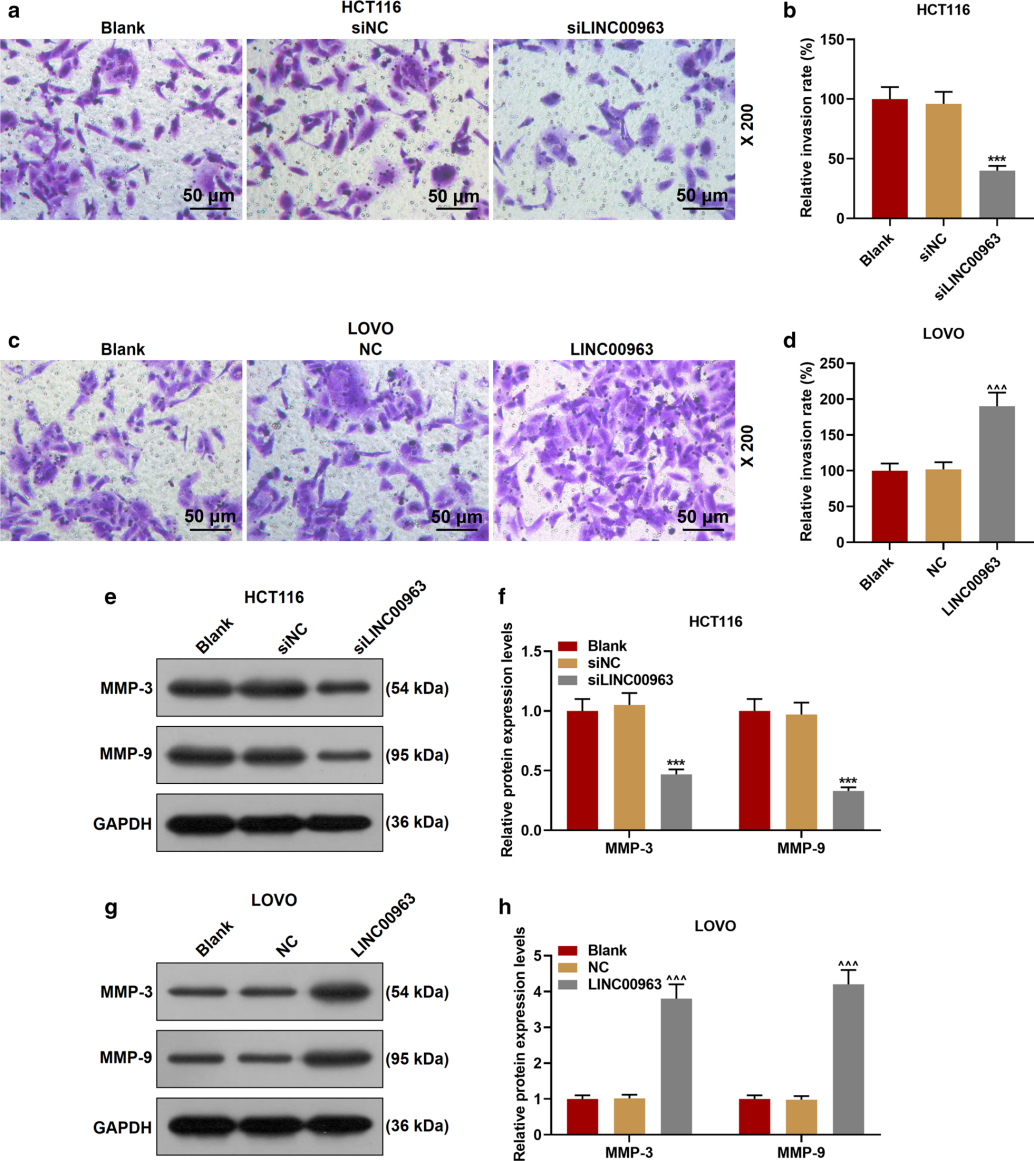
Silencing LINC00963 inhibited colon cancer cell invasion and migration-related protein expression, and overexpression of LINC00963 had the opposite effect. **a**–**d** The effect of overexpression or silencing of LINC00963 on colon cancer cell invasion was examined by Transwell. **e**–**h** LINC00963 overexpression promoted the expressions of MMP-3 and MMP-9, and silencing LINC00963 inhibited the expression of MMP-3 and MMP-9. The experiment was repeated three times independently. GAPDH served as a control. ^***^P < 0.001 vs siNC; ^^^^^*P* < 0.001vs NC

The LINC00963 overexpression plasmid and siRNA silencing LINC00963 plasmid were transfected into the corresponding HCT116 cells and LOVO cells for examining the biological function of LINC00963 in CRC. As shown in Fig. 2a, b, the expression of LINC00963 in cells was down-regulated after the transfection of silencing LINC00963, but up-regulated after the transfection of overexpressed LINC00963, indicating that the plasmid was successfully transfected into the cells (*P* < 0.001). Functional experiments showed that the cell activity and the number of cell clones in the siLINC00963 group were lower and fewer than the siNCup. Moreover, the LINC00963 group had the opposite results to the siLINC00963 group (*P* < 0.01, Fig. 2c–h). Silencing LINC00963 caused cell arrest in G0/G1 phase, and S phase and G2/M phase were shorter. After overexpression of LINC00963, we observed the G0/G1 phase was shorter and S phase and G2/M phase were longer (*P* < 0.01, Fig. 3a–d). In addition, silencing LINC00963 inhibited cell migration, invasion, and MMP-3 and MMP-9 expressions, while overexpression LINC00963 promoted cell invasion, migration, and MMP-3 and MMP-9 expressions (*P* < 0.001, Figs. 3e–h, 4a–h).

### LINC00963 could target miR-532-3p to regulate HMGA2 expression

**Fig. 5:**
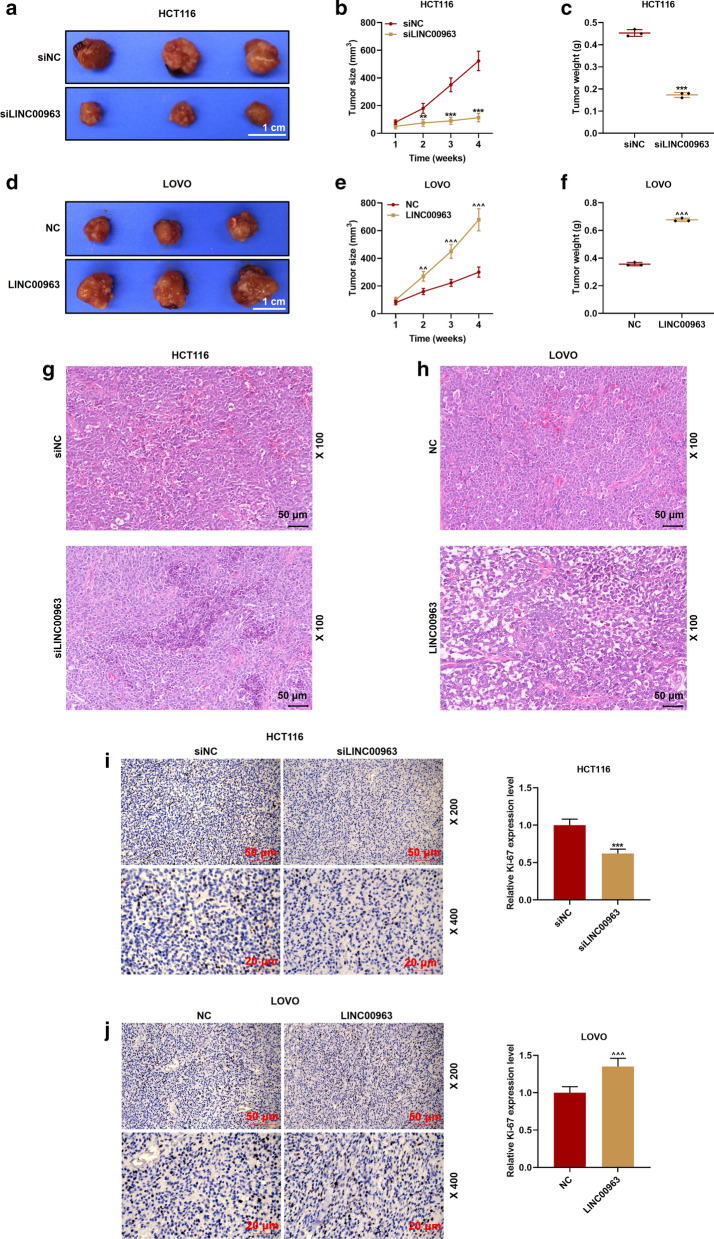
MiR-532-3p was the target miRNA of LINC00963. **a** StarBase and **b**, **c** dual-luciferase reporter gene assay were used to predict and verify that miR-532-3p binds to LINC00963. (D-E) RNA Immunoprecipitation was used to further verify that miR-532-3p binds to LINC00963. **f** The expression of miR-532-3p was down-regulated in colon cancer tissues (n = 50), and the results were detected by RT-qPCR. **g**, **h** The expression of miR-532-3p was negatively correlated with LINC00963, and the results were analyzed by Pearson correlation coefficient. **i**, **j** RT-qPCR was used to detect the expression of miR-532-3p in HCT116 cells or LOVO cells transfected with Blank, siNC, siLINC00963, and LINC00963, respectively. U6 served as a control. The experiment was independently repeated three times. RT-qPCR: real-time quantitative polymerase chain reaction. ^△△△^*P* < 0.001 vs Blank; ^***^P < 0.001 vs siNC; ^^^^^*P* < 0.001 vs NC; ^&&&^*P* < 0.001 vs Normal

StarBase prediction and dual-luciferase reporter gene analysis results confirmed that miR-532-3p could bind to LINC00963 (Fig. 5a–c). The RIP analysis further proved that miR-532-3p and LINC00963 were greatly enriched in the Ago2 antibody group compared with the control IgG antibody group (*P* < 0.001, Fig. 5d, e). Next, we examined the expression of miR-532-3p in CRC tissues, and found that miR-532-3p expression was down-regulated in CRC tissues (*P* < 0.001, Fig. 5f). The linear relationship between miR-532-3p and LINC00963 was shown by *Pearson* correlation coefficient, and it could be observed that miR-532-3p expression was inversely correlated with LINC00963 in normal tissues (*r* = − 0.307, *P* = 0.030, Fig. 5g) and CRC tissues (*r* = − 0.298, *P* = 0.035, Fig. 5h). RT-qPCR was further performed to validate that LINC00963 targeted to negatively regulate miR-532-3p, and overexpression of LINC00963 was found to inhibit miR-532-3p expression, and silencing LINC00963 promoted miR-532-3p expression (*P* < 0.001, Fig. 5i, j).

**Fig. 6:**
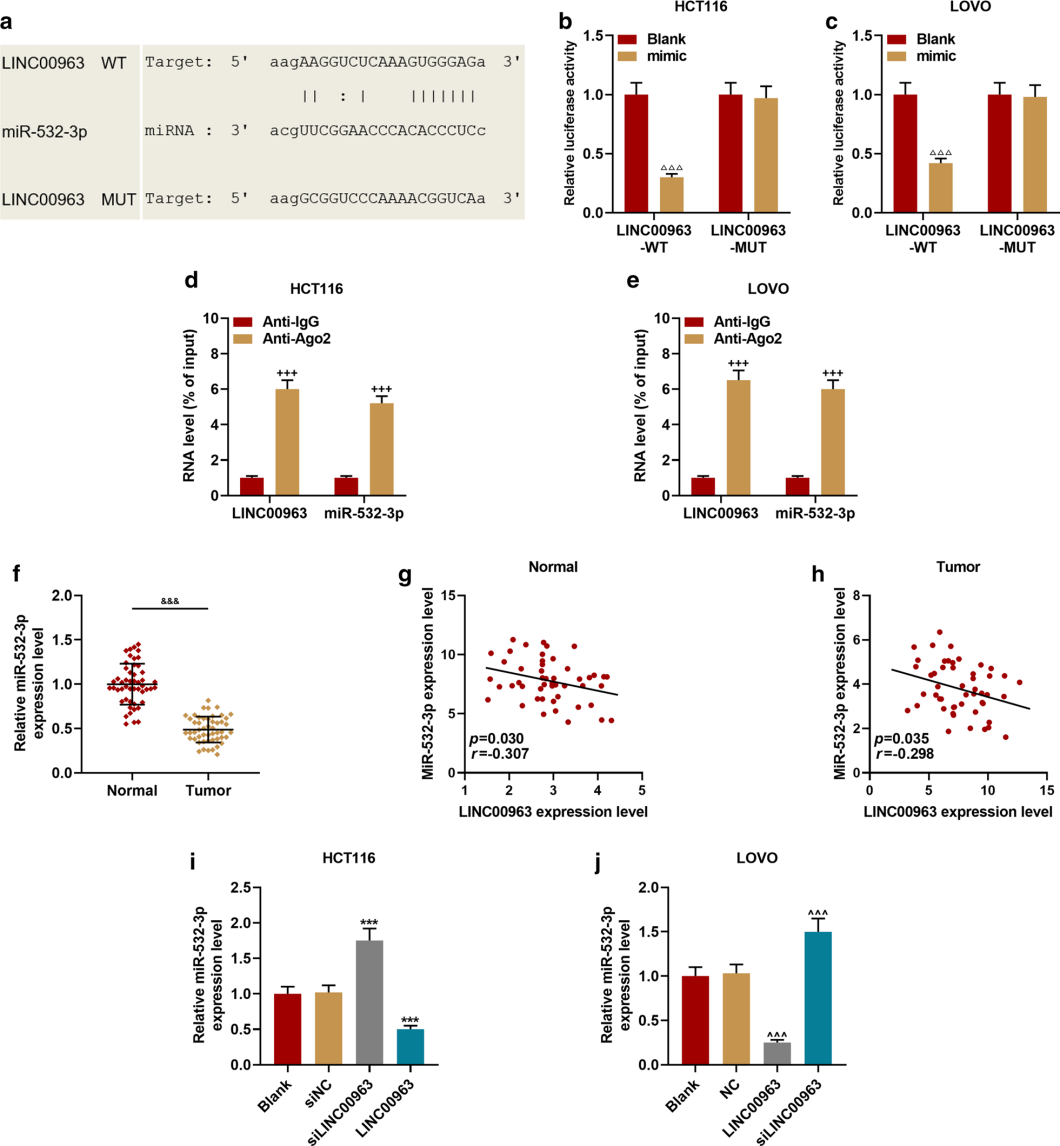
LINC00963 can target miR-532-3p to regulate HMGA2 expression. **a** The Venny map of miR-532-3p possible target gene, predicted by StarBase, Targetscan7.2, miRDB, miRBase database. **b** RT-qPCR was used to detect the expression of miR-532-3p in the Blank, siNC + IC, siLINC00963 + IC, siLINC00963 + I, siNC + I groups. **c** The expression of miR-532-3p in the Blank, NC + MC, LINC00963 + MC, LINC00963 + M, and NC + M groups was detected by RT-qPCR. **d**, **e** The effects of LINC00963 and miR-532-3p on the expressions of DDOST, HMGA2, MBD1, PISD, TARS were detected by RT-qPCR. U6 and GAPDH served as controls. The experiment was independently repeated three times. RT-qPCR: real-time quantitative polymerase chain reaction. ^*^P < 0.05, ^***^P < 0.001 vs siNC + IC; ^##^*P* < 0.01, ^###^*P* < 0.001 vs siNC + I; ^^^^^*P* < 0.001 vs siLINC + IC; ^&^*P* < 0.05, ^&&&^*P* < 0.001 vs NC + MC; ^△△△^*P* < 0.001 vs LINC + MC; ^+++^*P* < 0.001 vs NC + M

**Fig. 7:**
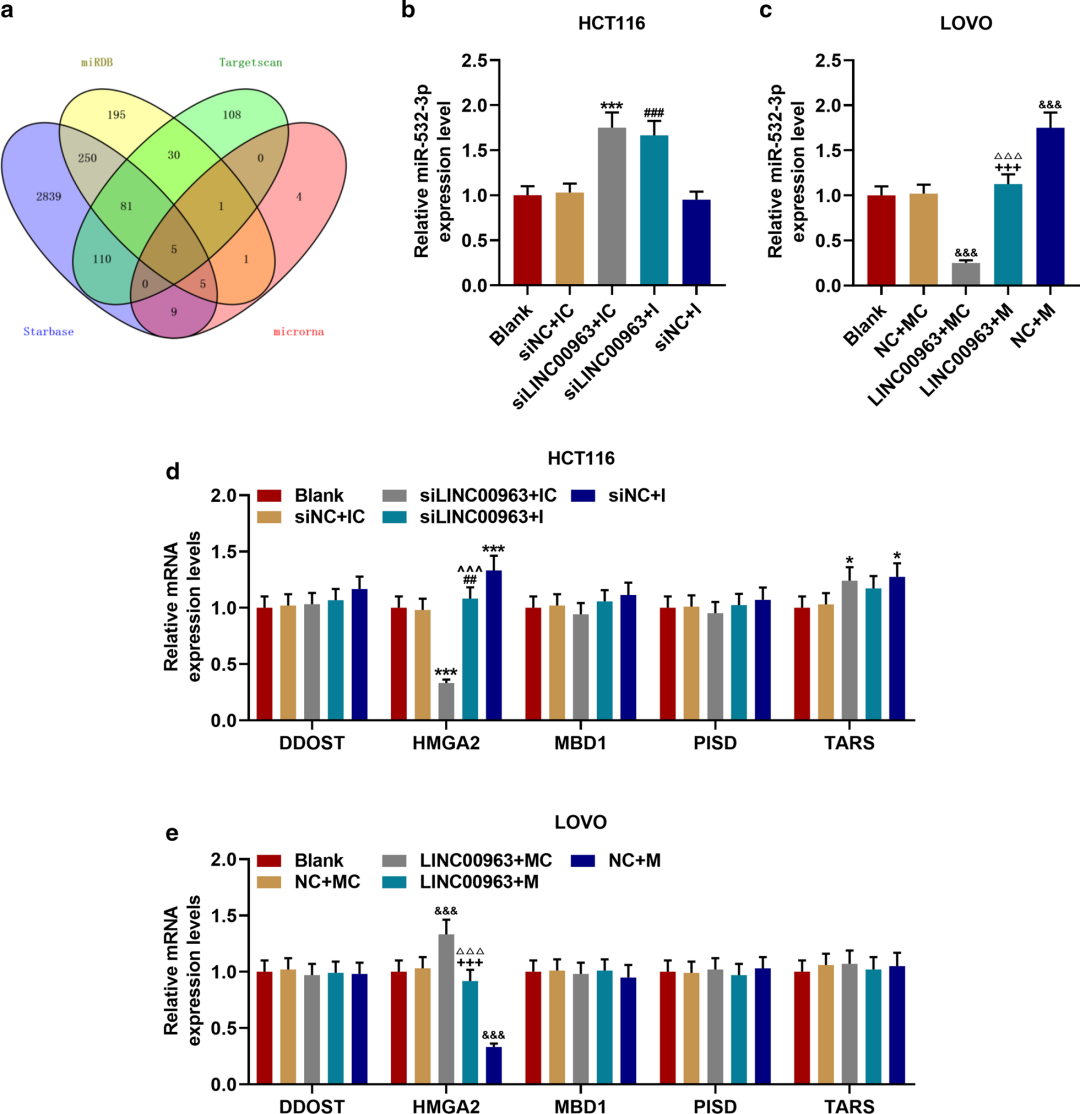
HMGA2 was high-expressed in colon cancer, and was negatively correlated with miR-532-3p and positively correlated with LINC00963. **a** TargetScan7.2 and (B-C) dual-luciferase reporter gene assay predicted and verified that HMGA2 binds to miR-532-3p. **d** HMGA2 was high-expressed in CRC. **e**–**h** The correlation between the expression of LINC00963, miR-532-3p and HMGA2 was analyzed by Pearson. ^△△△^*P* < 0.001 vs Blank; ^&&&^*P* < 0.001 vs Normal

**Fig. 8:**
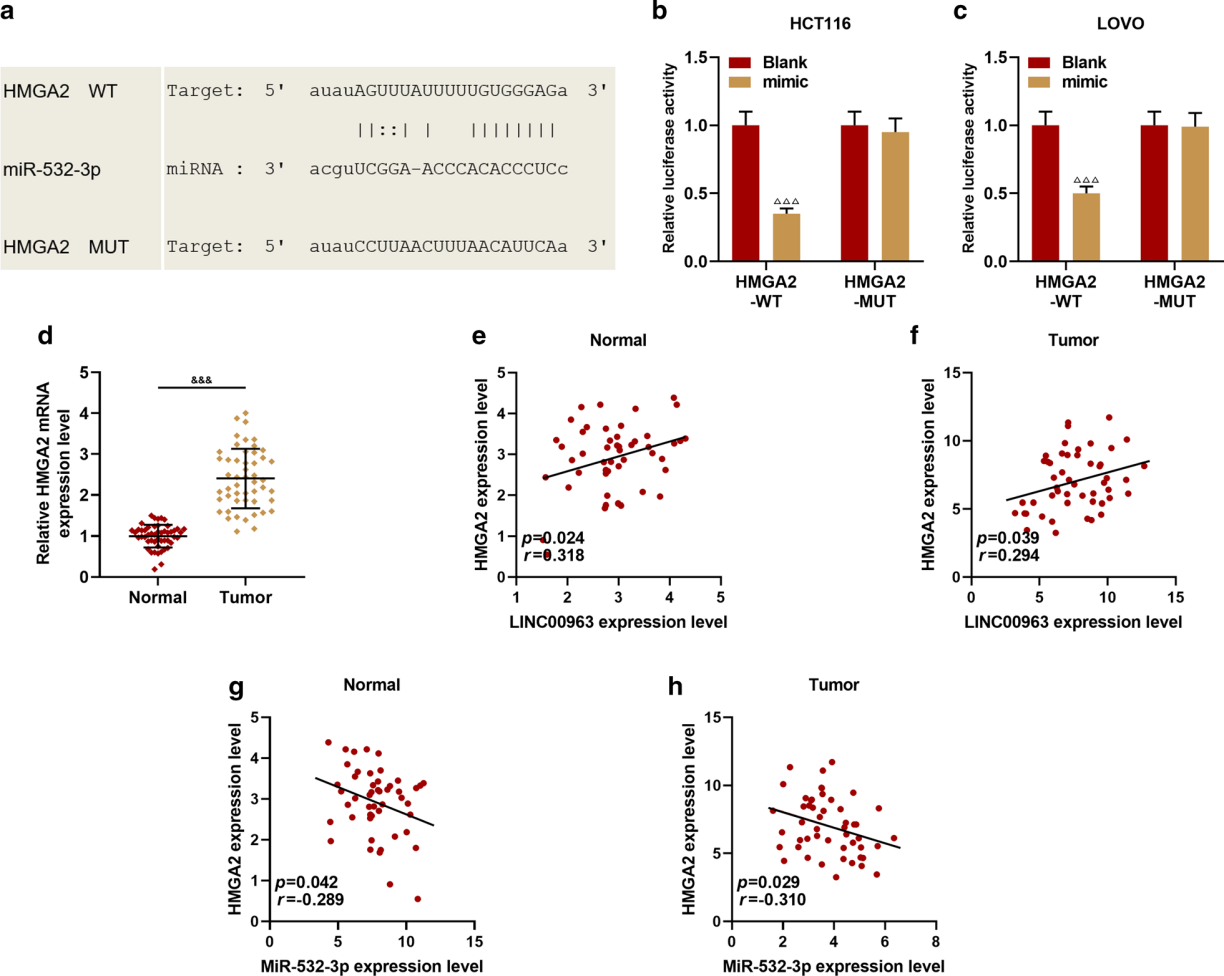
Effect of miR-532-3p targeting HMGA2 on proliferation of CRC cells**.** **a**, **b** siHMGA2 and miR-532-3p mimic inhibited HMGA2 expression, HMGA2 overexpression plasmid and miR-532-3p inhibitor had the opposite effects. **c**, **d** The effect of miR-532-3p and HMGA2 on cell migration was examined by CCK-8 experiment. **e**–**h** miR-532-3p inhibitor, HMGA2 overexpression promoted cell proliferation; miR-532-3p mimic, silencing HMGA2 inhibited cell proliferation. **i**, **l** The effects of miR-532-3p and HMGA2 on cell migration were detected by scratch experiments. ^**^P < 0.01, ^***^P < 0.001 vs IC + siNC; ^##^*P* < 0.01, ^###^*P* < 0.001 vs I + siNC; ^^^^^*P* < 0.001 vs IC + siHMGA2; ^&&^*P* < 0.01, ^&&&^*P* < 0.001 vs MC + NC; ^△△△^*P* < 0.001 vs MC + HMGA2; ^+++^*P* < 0.001 vs M + NC

In order to analyze the mechanism of LINC00963 and miR-532-3p in CRC, the potential target genes of miR-532-3p, namely DDOST, HMGA2, MBD1, PISD and TARS, were screened by bioinformatics analysis (Fig. 6a). Subsequently, the expressions of miR-532-3p and its target genes were determined. As shown in Figs. 6b–e, miR-532-3p expression was promoted by siLINC00963 and miR-532-3p mimic but inhibited by LINC00963, and miR-532-3p inhibitor slightly down-regulated miR-532-3p expression (*P* < 0.001). SiLINC00963 and miR-532-3p mimic inhibited the expression of HMGA2, while LINC00963 and miR-532-3p inhibitor produced opposite effects (*P* < 0.01). LINC00963 and miR-532-3p showed no obvious effect on the expressions of DDOST, MBD1, PISD and TARS. Therefore, the relationship between miR-532-3p and HMGA2 was further investigated, and we discovered that miR-532-3p targeted HMGA2 (Fig. 7a), moreover, the fluorescence activity of the HMGA2-WT + mimic group was lower than Blank group (*P* < 0.001, Fig. 7b, c). HMGA2 was high-expressed in CRC tissues (*P* < 0.001, Fig. 8d), positively correlating with LINC00963, while miR-532-3p was inversely correlated with HMGA2 (*P* < 0.05, Fig. 7e–h).

### MiR-532-3p regulated the biological characteristics of CRC cells through HMGA2

**Fig. 9:**
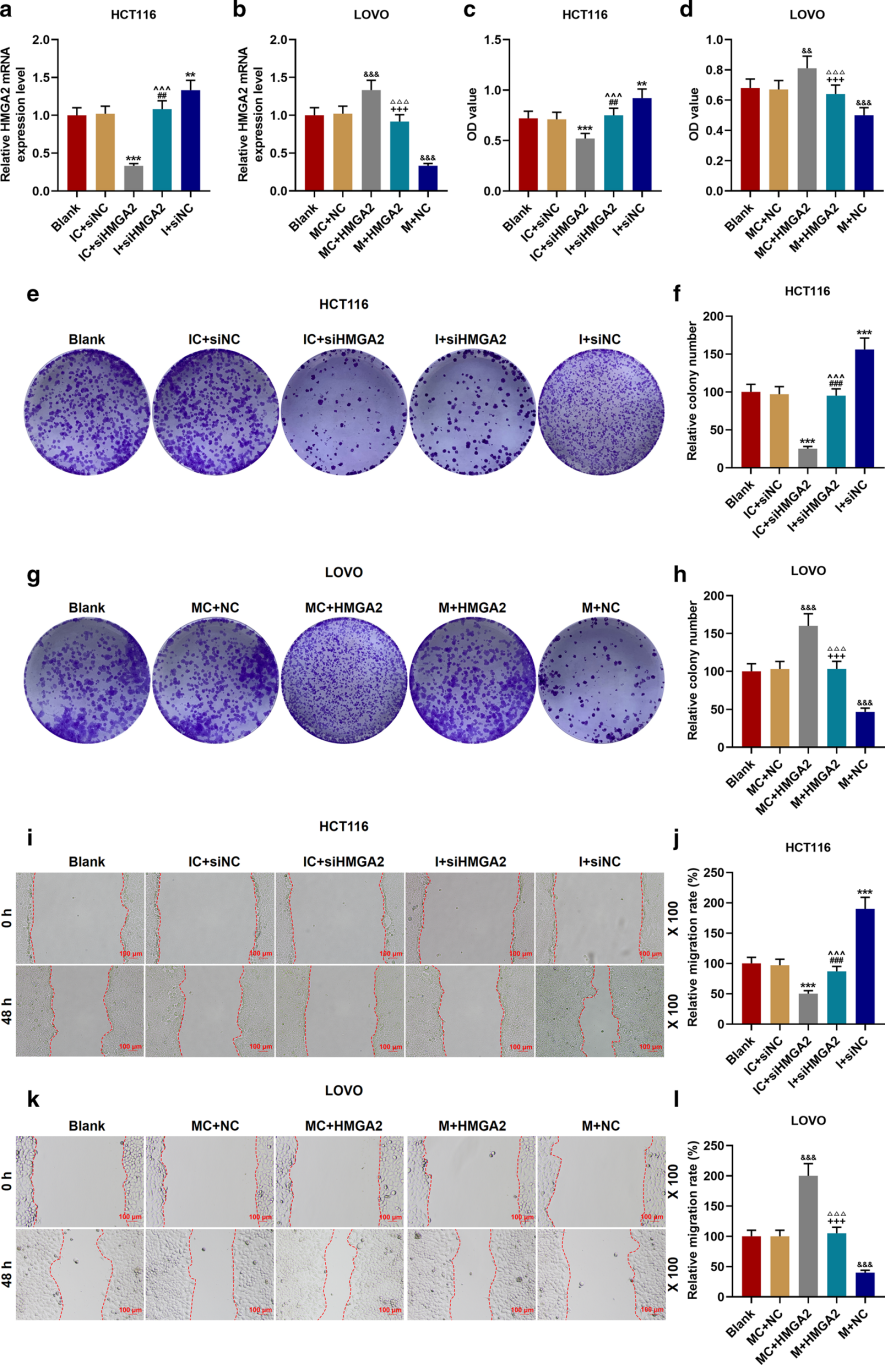
Effect of miR-532-3p targeting HMGA2 on invasion and migration-related proteins of CRC cells. **a**–**d** MiR-532-3p inhibitor and HMGA2 overexpression promoted cell invasion; miR-532-3p mimic and silent HMGA2 inhibited cell invasion. **e**–**h** The effects of miR-532-3p and HMGA2 on the expressions of MMP-3 and MMP-9 were determined by Western blot. GAPDH served as a control. The experiment was repeated three times independently. ^***^P < 0.001 vs IC + siNC; ^###^*P* < 0.001 vs I + siNC; ^^^^^*P* < 0.001 vs IC + siHMGA2; ^&&&^*P* < 0.001 vs MC + NC; ^△△△^*P* < 0.001 vs MC + HMGA2; ^+++^*P* < 0.001 vs M + NC

The expression of HMGA2 was down-regulated in IC + siHMGA2 group and up-regulated in I + siNC group, while the effects of IC + siHMGA2 group and I + siNC group were reversed in I + siHMGA2 group (*P* < 0.01, Fig. 8a). MiR-532-3p mimic inhibited the expression of HMGA2 previously increased by the transfection of HMGA2 overexpression plasmid (*P* < 0.001, Fig. 8b). As shown in Fig. 8c, d, miR-532-3p inhibitor increased cell activity, which was reversed by siHMGA2 (*P* < 0.01). Similarly, cell activity was inhibited by miR-532-3p mimic but such a results was reversed by HMGA2 overexpression (*P* < 0.01). In addition, functional tests and Western blot were performed to explore the roles of miR-532-3p and HMGA2 in CRC (Fig. 8e–l, Fig. 9a–h). Down-regulation of miR-532-3p promoted cell proliferation, migration and invasion, and expressions of MMP-3 and MMP-9, while siHMGA2 + I group significantly reversed the effect of I + siNC group (*P* < 0.001). Up-regulation of miR-532-3p inhibited cell proliferation, migration and invasion, and the expressions of MMP-3 and MMP-9, whereas HMGA2 + M group significantly reversed the effect of M + NC group (*P* < 0.001).

### Silencing of LINC00963 inhibited tumor growth, and overexpression of LINC00963 promoted tumorigenesis

**Fig. 10:**
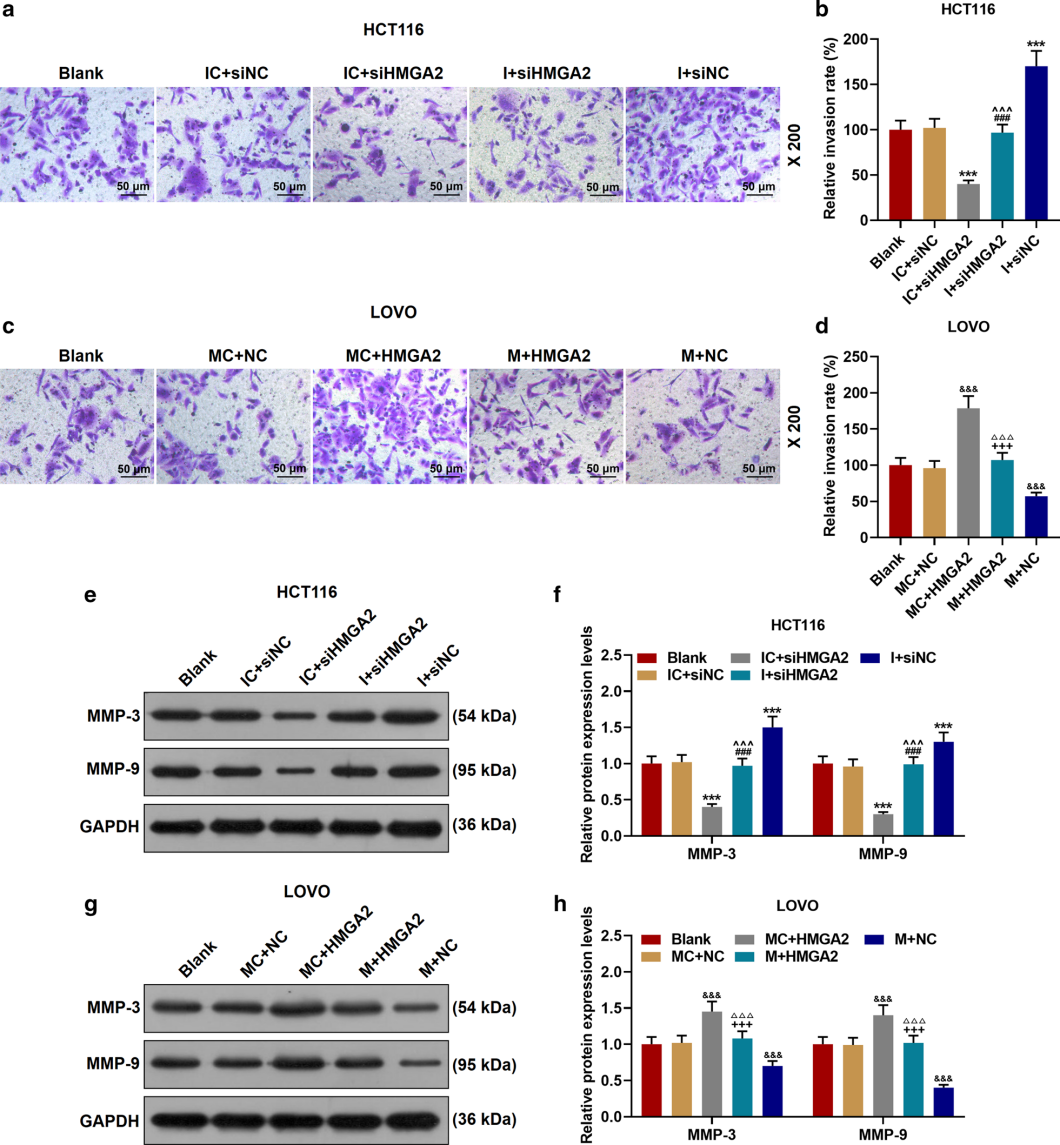
Effects of LINC0096 on tumor growth in nude mice. **a**–**f** Through observation and measurement of tumor volume and weight, we found that silencing LINC00963 inhibited tumor growth in the nude mice, and overexpression of LINC00963 promoted tumor growth. **g**–**j** Representative images of hematoxylin and eosin staining and immunohistochemistry of the tumor. ^**^P < 0.01, ^***^P < 0.001 vs siNC; ^^^^*P* < 0.01, ^^^^^*P* < 0.001vs NC

**Fig. 11:**
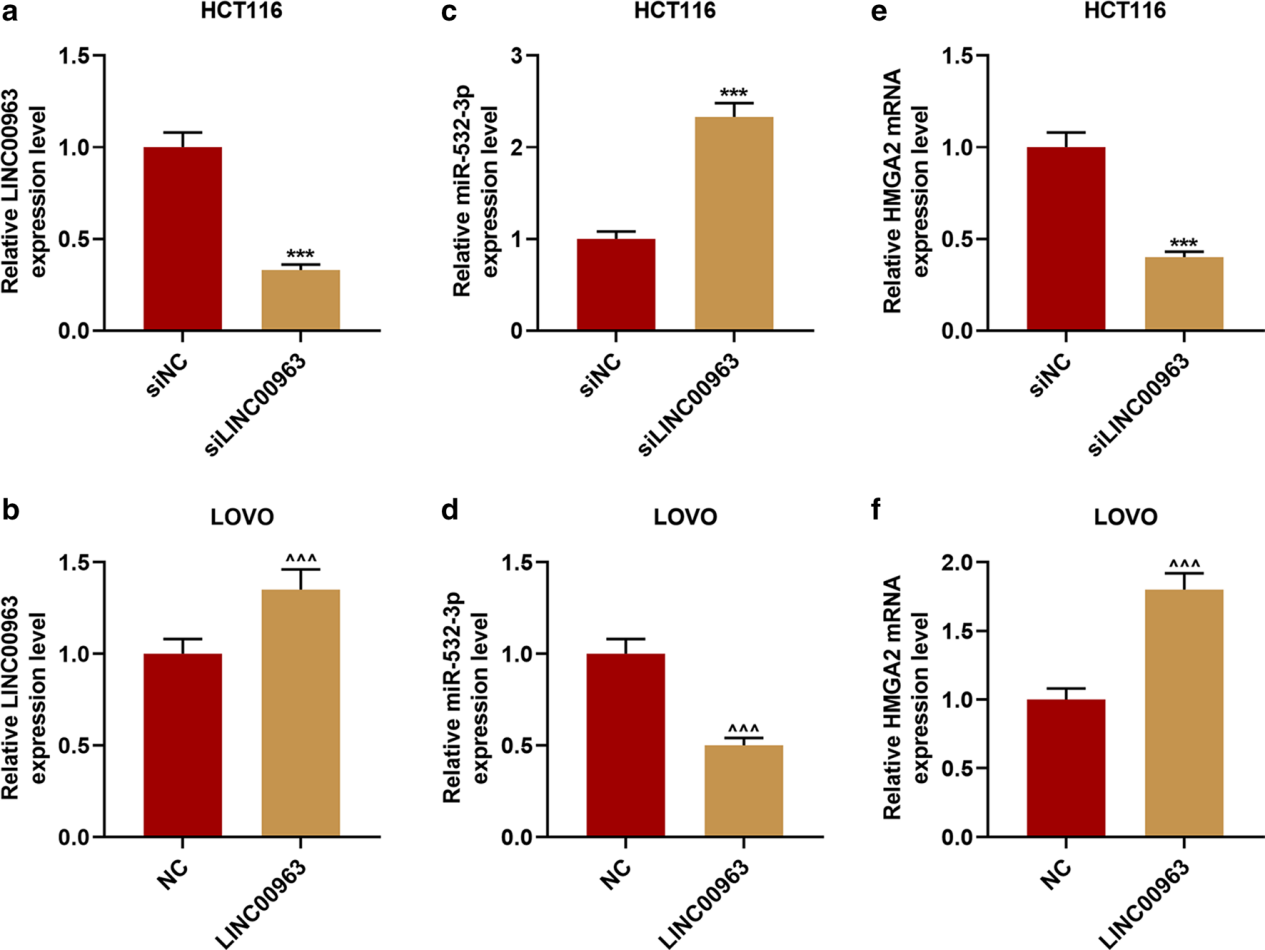
Effects of LINC0096 on miR-532-3p and HMGA2 expression in tumor. **a**–**f** The effects of silencing LINC00963 and overexpression of LINC00963 on the expressions of miR-532-3p and HMGA2 were determined by RT-qPCR. U6 and GAPDH served as controls. The experiment was independently repeated three times. RT-qPCR: real-time quantitative polymerase chain reaction. ^***^P < 0.001 vs siNC; ^^^^^*P* < 0.001 vs NC

The effect of LINC00963 in vivo was examined by carrying out a nude mouse tumorigenesis experiment. As shown in Fig. 10a–c, compared with the siNC group, the tumor size and weight were evidently reduced by siLINC00963 (*P* < 0.01), but LINC00963 overexpression promoted tumor growth. Specifically, the size of the tumor in the LINC00963 group was larger than that in the NC group, and the tumor weight was markedly heavier than that in the NC group (*P* < 0.01, Fig. 10d–f). HE staining was observed under the microscope, and comparing with the siNC group, we found that there were karyopyknosis and shape change in the siLINC00963 group, LINC00963 overexpression showed the opposite effect (Fig. 10g, h). Immunohistochemical staining experiments were further performed to detect Ki67 expression in the tumor tissues. Ki67 is a nuclear antigen associated with cell proliferation, and its function is closely related to the mitosis of cells [30]. As shown in Fig. 10g, h, silencing LINC00963 suppressed Ki67 expression (brown marker), while LINC00963 overexpression promoted Ki67 expression (brown marker) (*P* < 0.001, Fig. 10i, j). As shown in Fig. 11a–f, compared with the siNC group, siLINC009636 significantly inhibited the expression of miR-532-3p and promoted HMGA2 expression in the tumor. LINC009636 overexpression showed the opposite result (*P* < 0.001).

## Discussion

Exploring the molecular mechanism of CRC occurrence and discovering effective molecular therapeutic targets are crucial for elevating the survival rate of CRC patients [31, 32]. Abnormal expressions of lncRNAs are involved in the occurrence and development of CRC, which may provide directions for the diagnosis and treatment of CRC [5]. In this study, we found that the expression of LINC00963 was significantly up-regulated in CRC and was closely related to the poor prognosis of patients. Moreover, silencing LINC00963 was found to inhibit the growth, migration and invasion of CRC cells through regulating the expressions of miR-532-3p and HMGA2.

LINC00963 has been reported to be high-expressed in various cancers [13, 17]. For example, high-expressed LINC00963 is associated with poor prognosis and lymph node metastasis of ovarian cancer [33]. This study found that the expression of LINC00963 in CRC tissues and cells was markedly higher than that in the control group, and that the LINC00963 expression was also related to TNM staging and lymph node metastasis. The above findings revealed that LINC00963 may also have carcinogenic effects on CRC, which is consistent with the effects of LINC00963 on other cancers [33]. The progression of CRC is inseparable from cell proliferation. Several studies have confirmed that LINC00963 overexpression promotes cell proliferation, migration and invasion in vitro [34]. By performing in vitro experiments, LINC00963 was found to promote CRC cell proliferation, cycle progression, tumor growth, and up-regulated the expressions of MMP-2 and MMP-9. LINC00963. However, the results should be further verified by performing more related experiments.

LncRNAs can serve as oncogenes and tumor suppressors to target and regulate the expressions of miRNAs, thereby affecting disease progression [19]. Some scholars have examined the mechanism of action of LINC00963 in cancers, and found that LINC00963 relies on miR-204-3p to regulate fibronectin-1 to promote the proliferation and progression of osteosarcoma [34]. Furthermore, LINC00963 promotes tumorigenesis and radiation resistance of breast cancer by antagonizing miR-324-3p and inhibiting ACK1 expression [35]. In addition, LINC00963 regulates the progression of cutaneous squamous cell carcinoma through the miR-1193/SOX4 axis [36]. However, the molecular mechanisms of downstream of LINC00963 in CRC have not been explored. Our experiments found that LINC00963 was up-regulated in CRC tissues. LncRNAs play a regulatory role by communicating with DNAs, mRNsA, ncRNAs and proteins, and are involved various cancer-related cell processes [5]. Therefore, combined with previous studies, we found that LINC00963 was up-regulated in CRC, which was associated with its role as a tumor pro-oncogene in promoting proliferation and growth of cancer cells. Our experiments revealed for the first time that LINC00963 promotes CRC tumorigenesis through the miR-532-3p/HMGA2 axis.

MiR-532-3p plays an important part in a variety of diseases [37]. Jiang et al. showed that miR-532-3p inhibited the proliferation, invasion and migration of NSCLC cells through FOXP3 [38]. Ectopic expression of miR-532-3p significantly attenuated the malignant phenotype of the renal cell carcinoma cell line [39]. Study also found that miR-532-3p affects the malignant behaviors of tongue squamous cell carcinoma by targeting the downstream gene CCR7 [40]. In conclusion, the results indicated that miR-532-3p may have a targeted therapeutic effect on CRC. In this study, up-regulation of miR-532-3p inhibited cell activity, migration and invasion, reduce the number of clones, and down-regulated the expressions of MMP-2 and MMP-9, which is consistent with a previous study.

MiRNAs can form a gene silencing complex with other proteins [41]. The complex mainly recognizes the target by specifically binding the miRNA "seed sequence" to the 3 'UTR of the target mRNA or the complementary sequence of the coding sequence, thereby suppressing the effects of target mRNA on promoting tumor progression [41, 42]. We found that human high mobility group A2 (HMGA2) was the target gene of miR-532-3p. HMGA2, which has been observed to be able to affect a variety of biological processes, is a transcription factor without transcription function [43]. HMGA2 is mainly overexpressed in gastrointestinal tumors, including in CRC [44]. Accumulated evidence indicated that high-expressed HMGA2 is predictive of tumor progression, poor prognosis and adverse side effects of treatment [45]. The rescue experiment in this study showed that overexpression of HMGA2 can reverse the effect of miR-532-3p mimic on the biological characteristics of CRC cells. Furthermore, by performing in vivo experiments, up-regulating and down-regulating LINC00963 expression, we have fully demonstrated that LINC00963 has a cancer-promoting effect on CRC. It should also be noted that there are some limitations in our study. The sample size was small, also apoptosis-related mechanism, the function of HMGA2 in a xenograft model in the mice has not been examined.

## Conclusion

In conclusion, high-expressed LINC00963 is associated with poor prognosisof CRC through exerting carcinogenic activity of CRC via the miR-532-3p/HMGA2 axis. Thus, LINC00963 has the potential to be a therapeutic target for CRC.

## Supplementary information

**Additional file 1. Original images for Figure 4E blots**

**Additional file 2. Original images for Figure 4G blots**

**Additional file 3. Original images for Figure 10E blots**

**Additional file 4. Original images for Figure 10G blots**

## Data Availability

The analyzed data sets generated during the study are available from the corresponding author on reasonable request.

## Abbreviations

CRC: Colorectal cancer
MMPs: Matrix metalloproteinases
lncRNA: Long intergenic non-coding RNA
HCC: Hepatocellular
CCK: Cell Counting kit
IHC: Immunohistochemistry
HMGA2: High mobility group A2
RT-qPCR: Real-time quantitative polymerase chain reaction
HE: Hematoxylin and eosin
RIP: RNA Immunoprecipitation
