# Supplementary figures and images for: LINC00963 affects the development of colorectal cancer via MiR-532-3p/HMGA2 axis
Source: Cancer Cell Int. 2021 Feb 3;21:87. doi: 10.1186/s12935-020-01706-w (PMC7860506; doi:10.1186/s12935-020-01706-w)

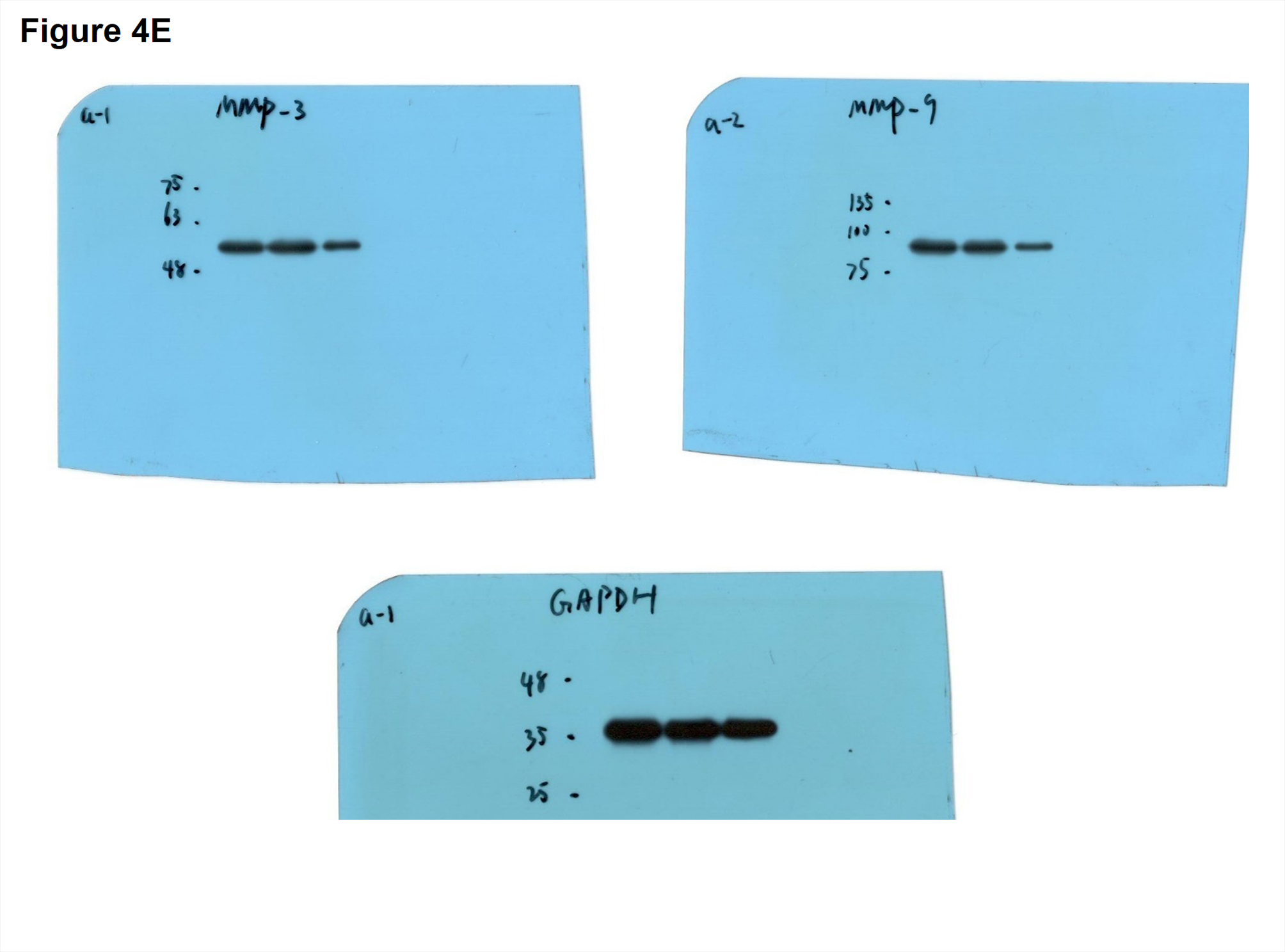

Supplement: Supplementary file 1 — Additional file 1. Original images for Figure 4E blots [file 12935_2020_1706_MOESM1_ESM.tif]

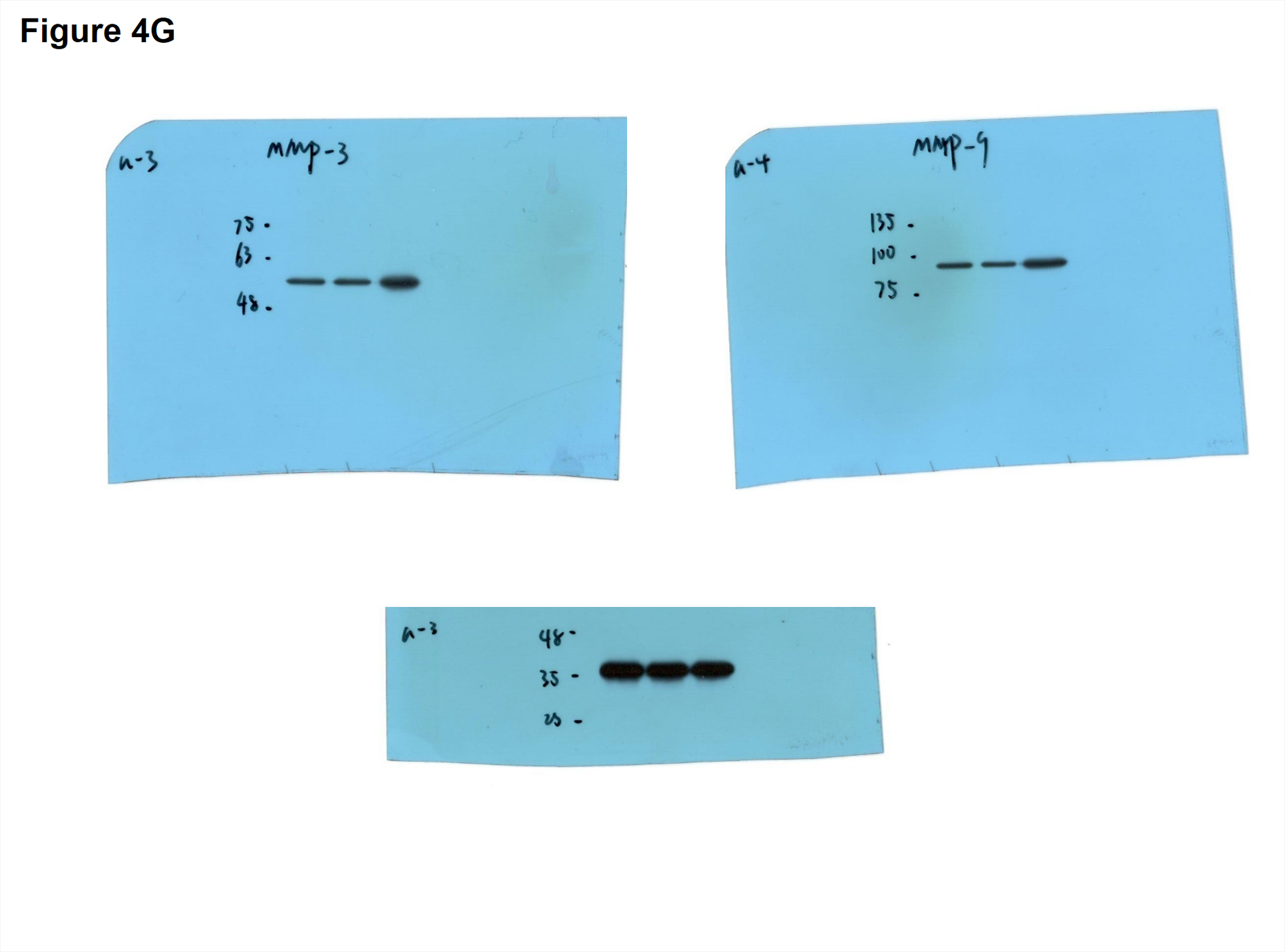

Supplement: Supplementary file 2 — Additional file 2. Original images for Figure 4G blots [file 12935_2020_1706_MOESM2_ESM.tif]

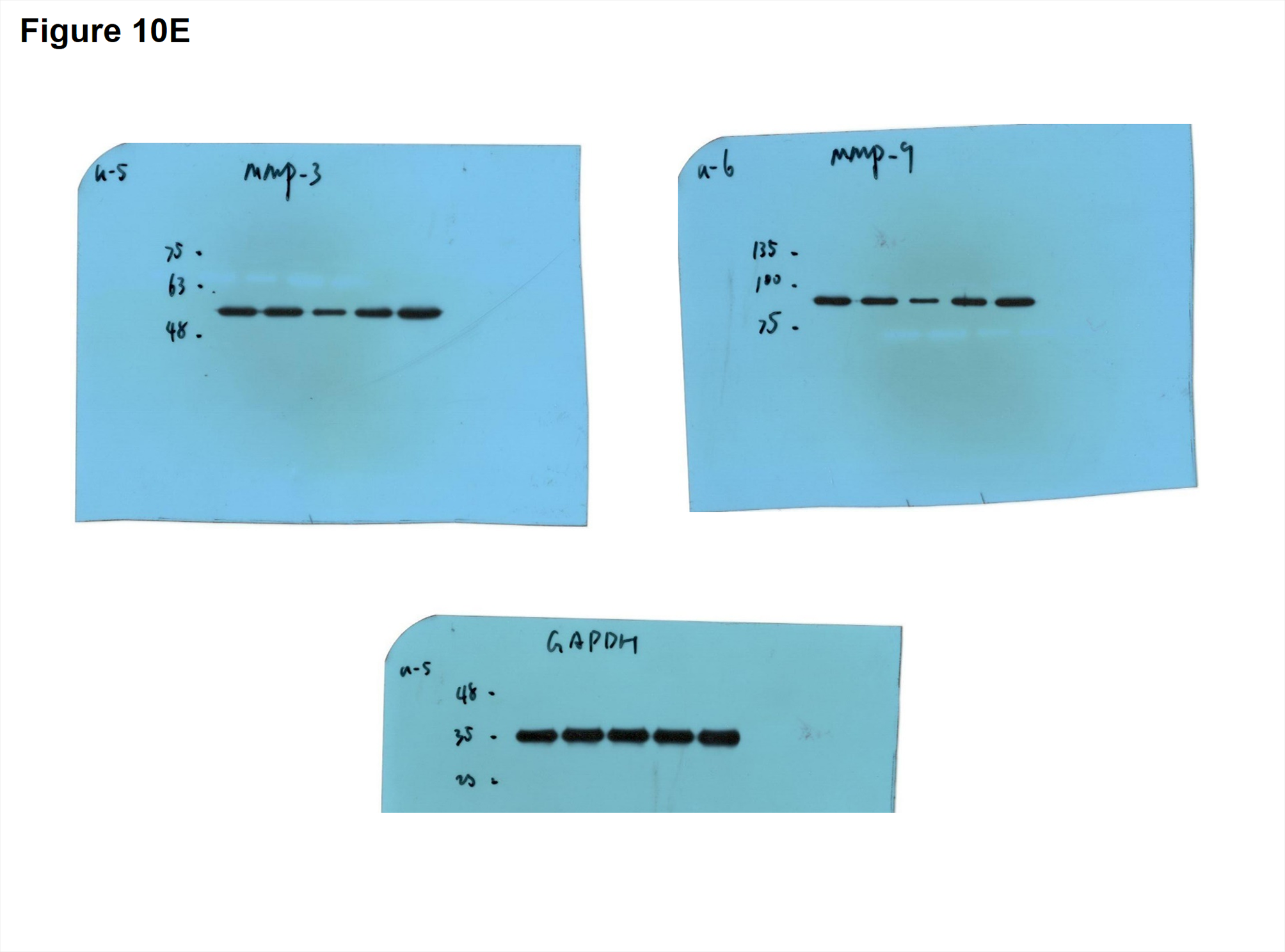

Supplement: Supplementary file 3 — Additional file 3. Original images for Figure 10E blots [file 12935_2020_1706_MOESM3_ESM.tif]

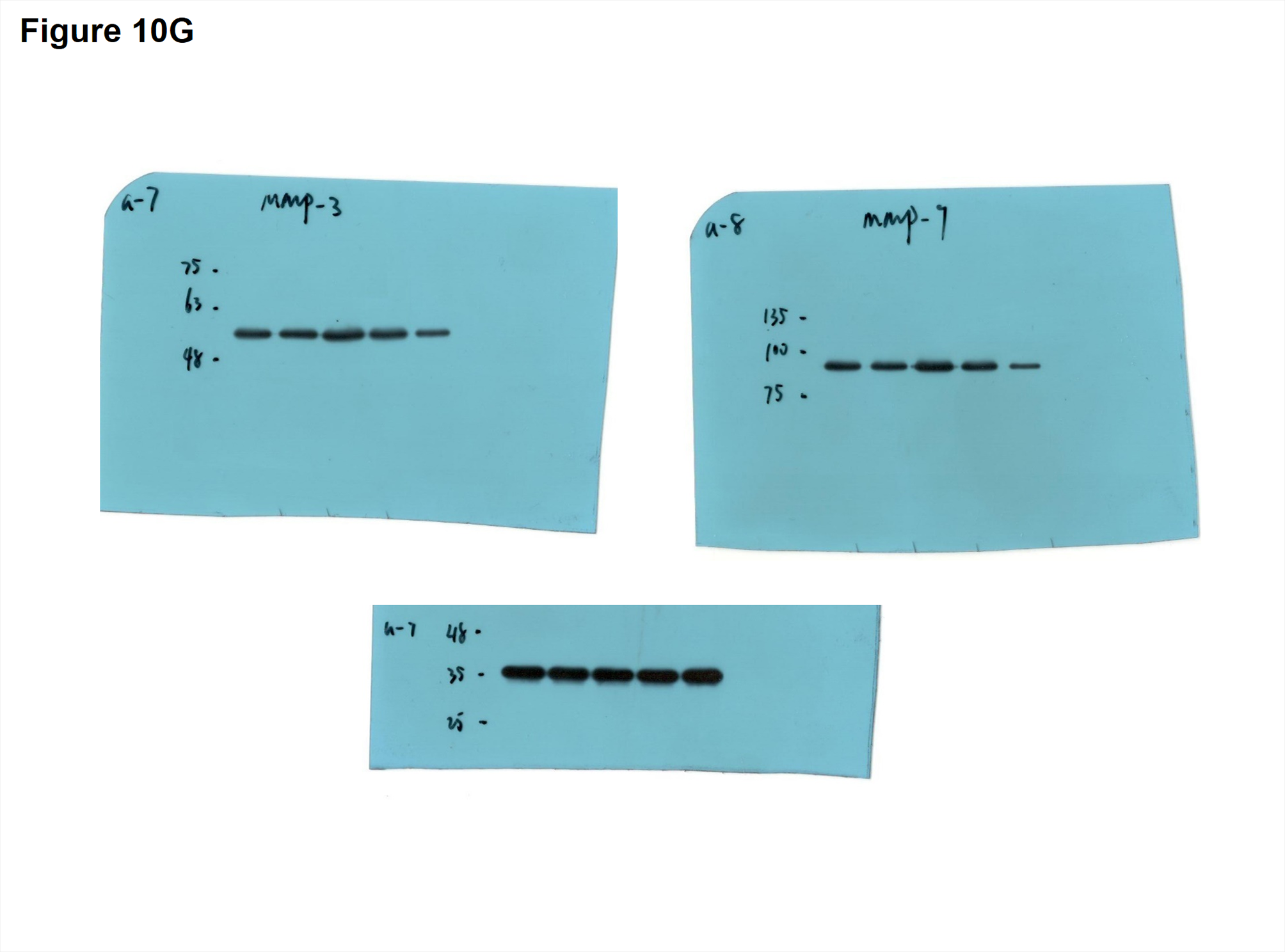

Supplement: Supplementary file 4 — Additional file 4. Original images for Figure 10G blots [file 12935_2020_1706_MOESM4_ESM.tif]
